# Correlation between timed up and go, usual gait speed and dizziness handicap inventory in elderly with vestibular disorders: a retrospective and analytical study

**DOI:** 10.1186/s40945-020-00083-x

**Published:** 2020-07-08

**Authors:** Daniel Héctor Verdecchia, Agustina Maria Monzón, Valentina Urbina Jaimes, Laercio da Silva Paiva, Fernando Rocha Oliveira, Tatiana Dias de Carvalho

**Affiliations:** 1 Departamento de Ciencias de la Salud, Kinesiología y Fisiatría, Universidad Nacional de La Matanza, Florencio Varela 1903, San Justo, B1754JEC Buenos Aires, Argentina; 2 Universidad Maimónides, Kinesiología Y Fisiatría, Ciudad Autónoma de Buenos Aires, Argentina; 3 Laboratório de Epidemiologia e Análise de Dados do Departamento de Saúde da Coletividade, Centro Universitário Saúde ABC, Santo André, SP Brazil; 4 Departamento de Epidemiologia, Faculdade de Saúde Pública da Universidade de São Paulo, São Paulo, SP Brazil

**Keywords:** Aged, Accidental falls, Rehabilitation, Vestibular diseases, Vestibular function tests

## Abstract

**Background:**

This study was done to verify the associations between the usual gait speed (UGS), the Timed Up and Go test (TUG), and the perception of disability in elderly vestibular patients and to identify factors associated with TUG results.

**Methods:**

This was a descriptive, analytical, and retrospective study that used data from the clinical records of vestibular patients aged 65 years or older at a rehabilitation service in Buenos Aires, Argentina. The records were examined for the following information: sex, age, type of vestibular disorder, dizziness handicap inventory (DHI) score and performance in the TUG and UGS tests before treatment. Pearson’s or Spearman’s correlation coefficient was used depending on the distribution of data. Age and the DHI were factored into multiple linear regression models in order to model the tests. A Receiver Operating Characteristic (ROC) curve was used to analyze the predictive power of age, the DHI total, and the UGS for the sample’s TUG results. The level of significance was 5%.

**Results:**

We evaluated 118 clinical records, of which 26 were excluded due to incomplete information, leaving data from 92 vestibular patients (73 females; 78.3 ± 5.8 years old). Unilateral vestibular hypofunction and Benign Paroxysmal Positional Vertigo presented the highest prevalence. The total score and the DHI domains showed a significant association with the TUG and UGS values. The age-adjusted DHI had a low predictive power for these same values.

**Conclusions:**

The total score and DHI domains have a significant association with the TUG and UGS values for elderly adults with vestibular disorders. The age-adjusted DHI has a low predictive power for TUG and UGS values.

## Background

Elderly persons with vestibular diseases often complain of dizziness, balance impairments, and visual – or gaze – disturbances [1, 2]. All of these are known risk factors for falls, which occur in up to 32% of individuals aged 65 to 74 years and 51% of those over the age of 85 [3]. Falls impact the physical, psychosocial, economic, and family life of these people and the resulting lesions range from small abrasions to fractures that are particularly common in osteoporotic bone [4].

The detection of the fall risk in persons with vestibular and balance dysfunction is a subject of great concern to health professionals [5]. Numerous simple and composite physical performance tests are used to screen for this risk among older adults and include the usual gait speed (UGS) and the Timed Up and Go (TUG) [6]. In addition, some questionnaires may be helpful for perceived disability, such as the Dizziness Handicap Inventory (DHI) [5, 7, 8].

The DHI is a self-assessment inventory designed to evaluate the precipitating physical factors associated with dizziness as well as the functional and emotional consequences of disorders of the vestibular system. It provides information that is useful for planning and assessing therapy [7]. The TUG test is simple, easy and quick to administer and requires no special equipment [9]. It is a test of balance that is commonly used to examine functional mobility in frail, community-dwelling, elderly adults and vestibular patients [5, 9, 10] and it has been shown that it is reliable between raters (intra-class correlation coefficient and Test-Retest reliability, ICC = 0.99) in hospital day patients [9]. It has also been shown to have significant correlations with the Berg Balance Scale (*r* = − 0.76), the Barthel Index (*r* = − 0.48), and the Tinetti Mobility Index (*r* = − 0.74) [11].

The UGS has been recommended as an appropriate measure for evaluating functional limitations [12]. It has good predictive power for such adverse health results as multi-morbidity, disability, and death [13, 14] and is a component of the Short Physical Performance Battery [15] in addition to being a tool for the evaluation of the physical performance of the lower limbs of older adults [16]. In vestibular patients, good correlations have been found between the TUG and the Four-Square Step Test, or FSST (*r* = 0.69 *p* < 0.01); between the TUG and the Dynamic Gait Index, or DGI (*r* = 0.56 *p* < 0.01); between the UGS and the FSST (*r* = 0.65 *p* < 0.01); and between the UGS and the DGI (*r* = 0.82 *p* < 0.01) [17]. In a study by Whitney et al. [5], the patients with the greatest perception of disability due to dizziness were the most functionally compromised and had a higher average TUG score than the mild or moderate groups, though the degree of association between both variables was not reported.

Simple tools for the detection of fall risk are essential for initiating treatment or prescribing precautions to minimize this risk [5], especially when it is possible to choose the best option for evaluating this condition. However, we have not found much information about the association between these three methods of assessment. Scientific evidence is confusing, because in some studies the TUG has been correlated with the UGS and with other tests [9], while in others, the TUG and the UGS have similar discriminative power for predicting the overall difficulty of activities of daily living [18–20]. In addition, there are studies which suggest that, unlike the TUG score, the UGS score can reflect the fine motor control ability of frail elderly adults [21].

It has been hypothesized that there may be a strong association between the gait speed and the TUG, a moderate association between the gait speed and the DHI, and also between the TUG and the DHI. It has been hypothesized as well that the age-adjusted DHI serves to predict the TUG and UGS values. The objectives of this study, then, were to verify the associations between the UGS and the perception of disability, and between the TUG test and the same perception in elderly people with vestibular disorders, and to identify factors associated with TUG results.

## Methods

### Data collection

This is a descriptive, analytical and retrospective study that uses data from the clinical records of vestibular patients aged 65 years or older at a rehabilitation service in Buenos Aires, Argentina. The records were examined for the following information: sex, age, type of vestibular disorder, DHI score and performance on the TUG and the UGS before treatment.

Incomplete records were the basis for exclusion. Both the rehabilitation clinic and the patients signed consent forms authorizing all procedures in accordance with The Code of Ethics of the World Medical Association (Declaration of Helsinki).

### Timed up and go

To perform the TUG, subjects were given verbal instructions to stand up from a seated position on a chair, walk 03 m as quickly and as safely as possible, cross a line on the floor, turn around, walk back, and sit down. Those subjects who used a helping device when walking in the community were requested to use that device [22]. One practice performance was permitted before the measured performance [10].

### Usual gait speed

To rate the usual gait speed, we used the timed 10-meter walk test. Each patient was instructed to walk at a comfortable, normal pace for 10 m. Only the middle 6 m section was timed to eliminate the effects of acceleration and deceleration. The start and stop of the performance time coincided with the toes of the leading foot crossing the 2 m mark and the 8 m mark, respectively. The speed was calculated on the basis of these data by dividing the middle 6 m by the time (in seconds) required to walk them [23].

### Dizziness handicap inventory

The DHI is a 25-item tool used to help patients rate their self-perception of handicap from dizziness [5]. It is subdivided into functional (36 points), emotional (36 points), and physical domains (28 points), and ranges from zero (no perceived handicap) to 100 (the maximum perceived handicap) [9]. We have used the Argentine version of this questionnaire [24], which is a reliable and valid tool for quantifying self-perceived handicap resulting from vertigo, dizziness or unsteadiness and has high internal consistency (α = 0.87) and very high test-retest reliability for the total DHI score (intra-class correlation coefficient: 0.98) and its domains.

### Statistical analysis

We carried out a descriptive analysis of the data. The characteristics of the population are presented here by absolute and relative frequency, and the quantitative variables are presented by measures of central tendency and dispersion in accordance with the normality test (Shapiro-Wilk test).

To analyze the correlations of the TUG and the UGS with age and handicap perception (physical, functional, emotional, and the DHI total), we used Pearson’s or Spearman’s correlation coefficient, depending on the distribution of data. Correlations of *r* > 0.9 were rated as very strong; those of r from 0.71 to 0.9 were rated as strong; r from 0.51 to 0.70, moderate; from 0.31 to 0.50, weak; and from 0.00 to 0.30, negligible. The same parameters were considered for negative correlations [25]. Multiple linear regression models were used in which the “TUG” and “UGS” tests were dependent variables and age and the Dizziness Handicap Inventory were independent variables. To analyze the predictive power of age, the DHI total, and the UGS for the TUG in the sample, a Receiver Operating Characteristic (ROC) curve was used. The values presented were based on the highest sensitivity and specificity, as described by Medronho et al. [26]. The level of significance was 5%. The statistical program used was the Stata 12.0 version.

## Results

We evaluated 118 clinical records, of which 26 were excluded because of incomplete information. Data from 92 vestibular patients (73 females; 78,3 ± 5,8 years old), were used. Unilateral vestibular hypofunction (UVH) and Benign Paroxysmal Positional Vertigo (BPPV) presented the highest case prevalence. None of the patients used walking aids. Table 1 shows the population characteristics.

**Table 1:** Description of population characteristics according to sex and diagnosis

Variable	n	%
	Mean (SD)	Minimum - Maximum
**Age (years)**	78.3 (5.8)	65–91
Sex		
Female	73	79.4
Male	19	20.6
**Diagnosis**		
Unilateral vestibular hypofunction (UVH)	34	36.9
Benign paroxysmal positional vertigo (BPPV)	22	23.9
Nonspecific diagnosis	18	19.6
Multisensory dizziness síndrome	8	8.7
Bilateral vestibular hypofunction	5	5.5
Central vestibulopathy	3	3.3
Mixed vestibular disorder	2	2.2
**Self-reported falls in the previous 1 year, n (%)**	23	25

*SD* standard deviation

**Fig. 1:**
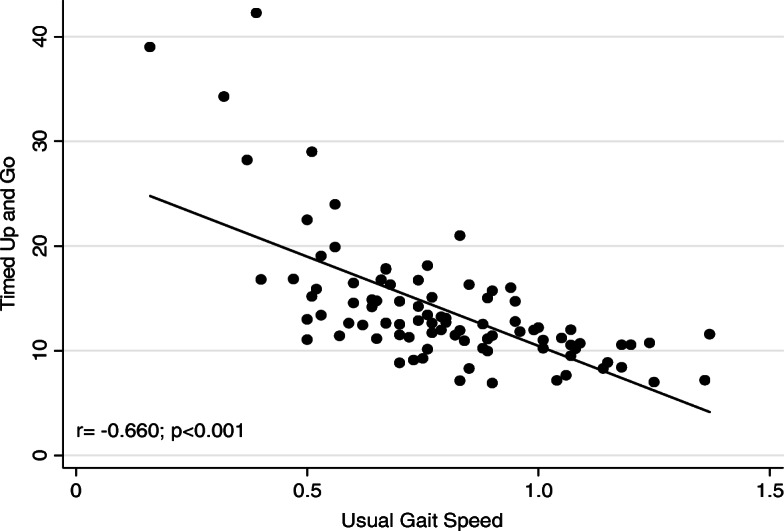
Correlation between timed and go and usual gait speed tests

Table 2 shows the TUG, UGS and Dizziness Handicap Inventory scores. The correlation data between the TUG and the UGS can be seen in Fig. 1. A negative and moderate, statistically significant correlation was observed between the tests (*r* = − 0.660, *P* < 0.0001).

**Table 2:** Timed Up and Go, usual Gait Speed and Dizziness Handicap Inventory scores

Variables	Mean (SD)	Median (p.25; p.75)	Minimum – Maximum
**Fall risk**			
UGS (m/s)	0.79 (0.23)	0.77 (0,64; 0,95)	0.16–1.37
TUG (s)	13.95 (6.14)	12.5 (10.7; 15.4)	6.9–42.26
**Handicap perception**			
Physical	15.67 (6.73)	16 (10; 21)	2–28
Functional	18.30 (9.23)	18 (10; 25)	2–40
Emotional	13.36 (9.12)	12 (6; 20)	0–36
DHI Total	47.34 (21.34)	45 (30; 65)	12–92

*SD* standard deviation, *p.25-p.75* Percentile 25–75, *UGS* Usual Gait Speed, *TUG* Timed Up and Go

**Fig. 2:**
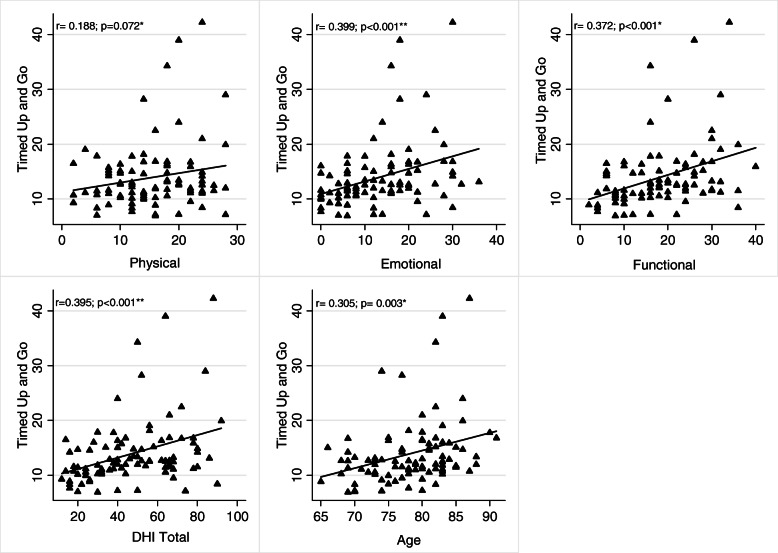
Correlation TUG with Physical, Functional, Emotional, DHI total and age. *Pearson test; **Spearman test; TUG: Timed Up and Go test; DHI - Dizziness Handicap Inventory. Significantly different *p* < 0.05

**Fig. 3:**
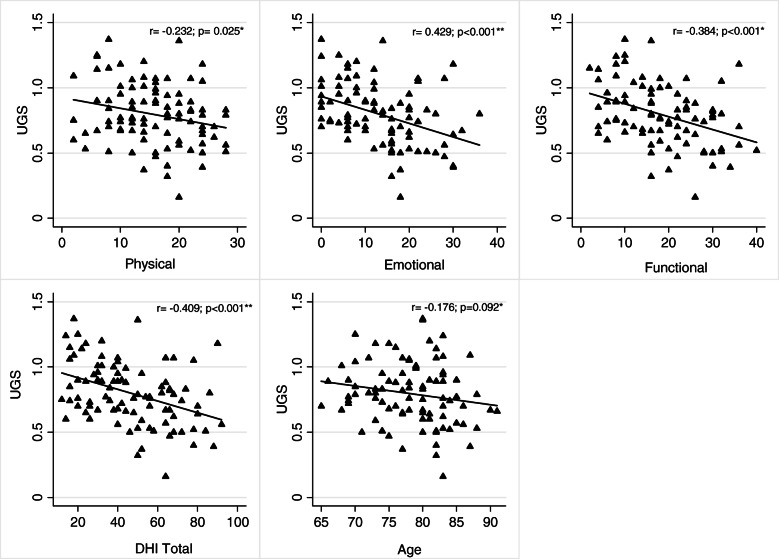
Correlation Gait Speed with Physical, Functional, Emotional, DHI total and age. *Pearson test); **Spearman test; UGS- Usual Gait Speed test; DHI - Dizziness Handicap Inventory. Significantly different *p* < 0.05

The correlations between the TUG and the domains and the total of the DHI and between the UGS and the three domains and the total of the DGI appear on Table 3 and in Figs. 2 and 3. The TUG test presented statistically significant correlations with the functional domain, the emotional domain, the DHI total, and age. The UGS presented statistically significant correlations with the physical domain, the functional domain, the emotional domain, and the DHI total.

**Table 3:** Correlation between TUG and UGS with Physical, Functional, Emotional, DHI total and age

Variable	Physical		Functional		Emotional		DHI total		Age	
	r	***p***	r	***p***	r	***p***	r	***p***	r	***p***
**TUG**	0.188	0.072	0.372	< 0.001*	0.399	< 0.001**	0.395	< 0.001**	0.305	0.003*
**UGS**	−0.232	0.025*	− 0.384	< 0.001*	−0.429	< 0.001**	− 0.409	< 0.001**	− 0.176	0.092

*Pearson test; **Spearman test; *TUG* Timed Up and Go test, *UGS* Usual Gait Speed, *DHI* Dizziness Handicap Inventory. Significantly different *p* < 0.05

**Fig. 4:**
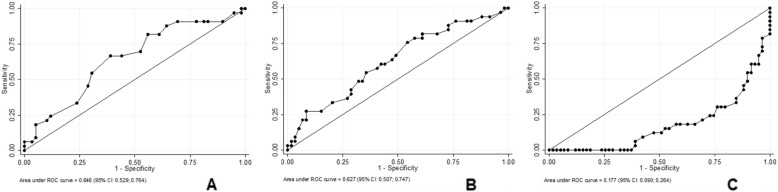
Effect of age (**a**), DHI (**b**) and usual gait speed (**c**) on the diagnostic ability of TUG. CI 95%: 95% confidence interval

Table 4 shows multiple linear regressions between the TUG as well as the UGS and the DHI adjusted for age. For the TUG, the model result demonstrated effect accounted for *r*^*2*^ = 0.2591. For the UGS, the model result demonstrated effect accounted for *r*^*2*^ = 0.1766. Figure 4 shows the effects of age (A: AUC: 0.646), DHI total (B: AUC: 0.627) and the usual gait speed (C: AUC: 0.177), respectively, on the diagnostic accuracy of the TUG.

**Table 4:** Multiple linear regression between TUG and UGS with DHI total and Age

Variable	β (CI 95%)	*p*	r2-adjusted
TUG			
DHI total	− 0.0008247 [− 0.0012125; − 0.000437]	< 0.001	0.2591
Age	− 0.0028904 [− 0.0043087; − 0.0014722]	< 0.001	
UGS			
DHI total	− 0.0045065 [− 0.0066126; − 0.0024003]	< 0.001	0.1766
Age	− 0.0074082 [− 0.0151115; 0.0002952]	0.059	

*CI 95%* 95% confidence interval, *TUG* Timed Up and Go test, *DHI* Dizziness Handicap Inventory, *UGS* Usual Gait Speed test

## Discussion

Our results showed a statistically significant negative and moderate correlation between the TUG and the UGS. The TUG presented statistically significant correlations with the functional domain, the emotional domain, the DHI total, and age; the UGS presented statistically significant correlations with the physical, functional, and emotional domains and with the DHI total. In addition, multiple linear regression indicated that the influence of age and the DHI total was 25% for the TUG and 17% for the UGS, with the age and the DHI total exerting more effect than the UGS on the diagnostic capability of the TUG.

As for the population characteristics, in our study there was a prevalence of females and the most common diagnoses were UVH and BPPV. Vestibular diseases affect both sexes and there is evidence to suggest that the patient’s sex may not affect rehabilitation outcomes [27]. The prevalence of UVH and BPPV coincided with previous studies showing that these were the most widely recognized vestibular disorders [28, 29].

What about the fall risk? The mean values of the UGS and the TUG were 0.79 m/s and 13.95 s, respectively, better values than other studies with similar populations [30]. In fact, it was observed that older adults who walked faster (higher UGS) had a lower risk of falls. Previous research had proposed TUG cut-offs of 12 s to reflect normal mobility [31], 14 s to reflect an increase in the falls risk [10], and 12.5 to 12.7 s to predict the onset of disability in daily living activities for older adults [19, 20].

In this study, the patients were instructed to walk at maximum speed, which would explain the shorter time in comparison with previous studies [30]. This test requires the ability to stand up and walk away and some patients may initially experience difficulty in this process, something which can also affect the total test time. These actions are very important in daily life, because, for independent mobility, one must be able to get in and out of a bed and a chair, on and off a toilet, and walk a few feet [9].

In addition, there was a statistically significant negative and moderate correlation between these tests (Fig. 1), something which was also observed in previous studies [7, 9]. Singh, et al. [7], examined the association between measured physiological fall risk and a battery of physical performance tests and found a moderate and significant correlation between the TUG and the UGS, since the higher the gait speed, the less time an individual takes to complete a distance. In one study [17] with 32 vestibular patients, the correlation between the TUG and the UGS was significant (0.66). This result was identical to the correlation we found between both tests. The time taken to complete the TUG test is strongly correlated to the level of functional mobility [10]. Adachi, et al. [32] found that specific parameters of the TUG test were associated with each clinical function test, and that the TUG test time used as an indicator of lower limb function and mobility had strong power of prediction for each motor function test. In our study, the TUG test presented a statistically significant correlation with the functional as well as the emotional domain and the DHI total.

We also found statistically significant correlations between the UGS and the physical, functional, and emotional domains and the DHI total. Zanotto et al. [33], when evaluating the association between the DHI screening version score and spatiotemporal gait parameters, indicated that DHI scores significantly improved the predictions of walking speed and other temporal parameters and that these predictions were more reliable than those based on differences in age, sex, and race/ethnicity. However, in one study on vestibular patients [17], no significant association was found between the TUG and the total DHI (rho 0.00) nor between the UGS and the total DHI (rho 0.22). This may be due to a smaller sample size and to the fact that the patients were younger than those who participated in our study. According to the multivariate analysis, the DHI questionnaire could only explain up to 25% of the results of physical tests like the TUG and the UGS. This shows that it is necessary to carry out these functional tests together with self-administered questionnaires. Although the DHI is a reliable tool and valid for measuring limitations on everyday activities and restrictions on social participation for patients with vestibular disorders [34], the sole use of self-administered questionnaires probably contributed in part to the deficiencies found in our patients, which is why we recommend including functional tests for this patient population.

In addition, age and the DHI total have a greater effect than the UGS on the diagnostic value of the TUG. The use of functional tests like the TUG and the UGS provide physiotherapists with more objective measures, and not only for the purpose of evaluating the functional status of patients and predicting future falls, but also for helping to choose the best exercises for their patients´ individual needs in order to reach the objectives of a vestibular rehabilitation program. Although we have found that age and the DHI affect the diagnostic ability of the TUG, in our study the UGS did not. We found no explanation for this last finding; we had expected the gait speed to affect the diagnostic ability of the TUG, since the patient has to walk for a great part of this test. We believe that administering the TUG at a maximum but safe gait speed and the UGS at a comfortable speed could have influenced the effect of the UGS on the diagnostic capability of the TUG.

The predominant number of women in the sample may well have been a limitation of the present study. However, previous studies confirm that vestibular disorders affect both genders, and that these show no differences in results [27]. Further research is necessary to indicate other factors that could predict the UGS and the TUG of elderly adults. To the best of our knowledge, this is the first study to verify the associations between the UGS, the TUG test, and the DHI, as well as to identify factors associated with TUG results in elderly people with vestibular disorders in South America. We hope that this information can be used clinically to better monitor patients, especially after rehabilitation programs.

## Conclusion

The total score and the DHI domains have a significant association with TUG and UGS values for older adults with vestibular disorders. The age-adjusted DHI has low predictive power for TUG and UGS values.

## Data Availability

Not applicable.

## Abbreviations

BPPV: Benign paroxysmal positional vertigo
DHI: Dizziness Handicap Inventory
GS: Gait speed
TUG: Timed Up and Go
VR: Vestibular rehabilitation
